# Student perspectives on competency-based portfolios: Does a portfolio reflect their competence development?

**DOI:** 10.1007/s40037-020-00571-7

**Published:** 2020-04-09

**Authors:** Andrea Oudkerk Pool, A. Debbie C. Jaarsma, Erik W. Driessen, Marjan J. B. Govaerts

**Affiliations:** 1 School of Health Professions Education (SHE), Maastricht University, Maastricht, The Netherlands; 2 Department of Educational Development and Research, Faculty of Health Medicine and Life Sciences, Maastricht University, Maastricht, The Netherlands; 3 Center for Education Development and Research in Health Professions (CEDAR), Faculty of Medical Sciences, University Medical Center Groningen, University of Groningen, Groningen, The Netherlands

**Keywords:** Portfolio, Assessment, Competency-based medical education, Medical students

## Abstract

**Introduction:**

Portfolio-based assessments require that learners’ competence development is adequately reflected in portfolio documentation. This study explored how students select and document performance data in their portfolios and how they perceive these data to be representative for their competence development.

**Methods:**

Students uploaded performance data in a competency-based portfolio. During one clerkship period, twelve students also recorded an audio diary in which they reflected on experiences and feedback that they perceived to be indicants of their competence development. Afterwards, these students were interviewed to explore the extent to which the performance documentation in the portfolio corresponded with what they considered illustrative evidence of their development. The interviews were analyzed using thematic analysis.

**Results:**

Portfolios provide an accurate but fragmented picture of student development. Portfolio documentation was influenced by tensions between learning and assessment, student beliefs about the goal of portfolios, student performance evaluation strategies, the learning environment and portfolio structure.

**Discussion:**

This study confirms the importance of taking student perceptions into account when implementing a competency-based portfolio. Students would benefit from coaching on how to select meaningful experiences and performance data for documentation in their portfolios. Flexibility in portfolio structure and requirements is essential to ensure optimal fit between students’ experienced competence development and portfolio content.

**Electronic supplementary material:**

The online version of this article (10.1007/s40037-020-00571-7) contains supplementary material, which is available to authorized users.

## Introduction

Portfolios are used to foster student development as well as to enable decision-making about competence achievement [1]. Portfolio-based assessments therefore require that the learner’s competence development is adequately reflected in the portfolio content. Students have a prominent role in collecting and documenting portfolio content. A review of the portfolio literature found some evidence for the content validity of portfolios [2]. A later study at the Cleveland Clinic Lerner College of Medicine demonstrated that, with monitoring from faculty, students are able to select evidence and document performance evaluations for summative decisions [3]. These studies did not, however, study portfolios used for competence assessment in clinical education.

In clinical settings, competency-based portfolios largely consist of workplace-based assessments (WBAs). WBAs have, however, been implemented with mixed success [4]. In most competency-based education programs, it is the responsibility of students to collect WBAs that provide evidence of their competence development or mastery of entrustable professional activities. Collecting meaningful WBAs can be difficult for several reasons. For example, students may strategically ask for assessments in situations in which they are confident about task performance and avoid assessments in situations when they feel less confident [5]. Furthermore, in the clinical workplace, the time available for assessment is often limited. Engaging in WBA is often perceived to take time away from patient care. Faculty may therefore struggle to schedule WBAs and students may hesitate to ask for direct observations and evaluations of task performance if they feel that faculty are too busy [6, 7]. Another issue impacting student willingness to initiate WBA is that a student may feel nervous and intimidated when observed [6].

These issues with WBA can potentially affect the content of workplace-based portfolios, as some competencies and task- or content-specific performances are likely to be underrepresented in the portfolio, whereas other information might be overrepresented. This would imply that the portfolio content may not always accurately reflect a student’s development and level of competence.

In competency-based assessment, it is essential that the portfolio content mirrors a student’s competence development to guide learning as well as to support high-stakes decision-making. Given the agentic role of students in the composition of the portfolio it is important to understand their perspective on the extent to which competency-based portfolios mirror their competence development. A better understanding of this is fundamental because clinical competency committees mainly base their assessments on the content of competency-based portfolios [8]. In order to further our understanding, this study explored the following two research questions:

1. How well do students think their portfolio reflects their competence development? and
2. How do students select and document their performance in a portfolio?

## Methods

In this study, we triangulated data from students’ audio diaries capturing day-to-day learning experiences, their competency-based portfolio content, and interviews with students.

### Setting

The study was set in the final 3 years of the 6‑year undergraduate medicine program of Maastricht University, the Netherlands. These final 3 years of the curriculum consist of clinical clerkships, a research project, and electives. Clinical clerkships typically last between 8–20 weeks depending on the discipline and the type of clerkship. The curriculum is designed according to the principles of competency-based education and programmatic assessment, using the CanMEDS competencies as an overarching framework [8]. The assessment program is supported by a web-based portfolio system in which students collect and reflect on evidence of their learning and development in each of the competency domains [9, 10].

At the start of their clerkship, the student’s competency-based portfolio only contains their learning plan. Over the course of the clerkship, the portfolio is filled with self-assessments, WBAs (mini-clinical examinations (mini-CEX), direct observation of procedural skills, field notes, multi-source feedback, case-based discussions), progress test results and reflections on their learning process. Students are responsible for collecting WBAs in different settings from various assessors in order to ensure broad sampling. Depending on the clerkship, students gather between 21 and 26 WBAs in total. Each portfolio comprises narrative feedback and competency ratings (i.e., poor, average, and good) for the competency domains.

Mentors support student learning. Students and mentors meet 3–4 times a year to discuss the student’s competence development and to formulate a new learning plan.

Annually, a clinical competency committee makes a formal pass-fail decision about the student’s competence development based on evidence in the portfolio and an advisory judgment from the mentor.

#### Participants

We sent students an invitation email explaining the goal and procedure of the study. Subsequently, the principal investigator (A.O.P.) visited the clerkship introduction days to invite students to participate. Twenty-one students within surgical, non-surgical and family medicine clerkships gave their informed consent and agreed to participate. Twelve students finished the study. Two students were in their final year and the others were fourth year students. Nine students decided to withdraw from the study because of the heavy workload associated with their clerkship. Their data were not included in the analysis in accordance with the informed consent form, which stated that their data would be deleted if they decided to withdraw their participation. Students who completed the whole procedure received €100 in gift vouchers.

#### Research procedure

We collected data between November 2016 and May 2017. The research procedure consisted of three steps.

##### Step 1

First, we wanted to have a better understanding of how students had experienced their development during the clerkship. Therefore, we asked students to record an audio diary twice a week during their clerkship using the audio recorder on their smartphone. The audio diary contained reflections on feedback and experiences that students perceived to be important and illustrative of their competence development. We used audio diaries because this enabled the students to regularly and instantly capture how they experienced their competence development process. The length of the recordings varied between 4–9 min. Standardized questions about their learning experiences prompted student reflections (Appendix 1 of the Electronic Supplementary Material). The students sent their audio files via email to A.O.P. The audio diaries were not part of the official portfolio procedure nor were they used in formal decision-making about student competence achievement.

##### Step 2

At the end of their clerkship the students granted the principal investigator access to their competency-based portfolio. A.O.P. compared the portfolio content with the content of the audio diary. Using content analysis, it was determined whether the main learning experiences and feedback captured in the audio diary were also documented in the portfolio and vice versa. Also, A.O.P. asked students to select two audio diary fragments that described experiences that were most illustrative for their development. The results from the comparative content analysis and the selected fragments served as a starting point for the interviews conducted in step 3.

##### Step 3

After the clerkship, A.O.P. conducted semi-structured one-on-one interviews with the students. Interviews lasted about one hour. The aim of the interview was to gain an understanding of the extent to which students thought the portfolio reflected their competence development during the clerkship. The audio fragments were used to stimulate students to recall those experiences and feedback that they had considered most important for their development. Students were encouraged to compare their audio diaries with evidence uploaded to the portfolio and to elaborate on the extent to which the portfolio captured their development. Furthermore, questions focused on how students had used their portfolios to document their competence development and which portfolio elements would provide them and others insight into their learning process and competence achievement. The final interview guide can be found in Appendix 2 of the Electronic Supplementary Material. All interviews were in Dutch, audiotaped and transcribed verbatim.

### Analysis

We analyzed the interview data using thematic analysis [11]. A.O.P and a research assistant (C.N.) coded the first two transcripts and developed an initial coding manual, on the basis of which another research assistant (A.B.) then coded the same transcripts again. Subsequently, A.O.P., and A.B. discussed the codes and themes and further refined the initial coding scheme. A.O.P., C.N. and A.B coded the remaining transcripts. After all transcripts were coded, the research team (A.O.P., M.G., E.D., and D.J.) discussed the key themes and conceptualizations that the students reported. Summaries of the discussions served as a basis for the further analysis of the transcripts by A.O.P and A.B. The research team met several times to further review and refine themes and define relations between themes in order to develop an understanding of how students compose their portfolio and think that their portfolio reflects their competence development. ATLAS.ti software v1.0.17 for Mac (Scientific Software Development GmbH, Berlin, Germany) was used to facilitate the data analysis.

## Results

The dynamic nature of competence development, students’ beliefs about the purpose of a portfolio, what information they considered valuable for assessors, and their strategies in feedback documentation inhibited the representativeness of the portfolio. Also, the portfolio structure influenced the documentation of evidence. These aspects will be further explained in this section.

### Snapshots of competence development

Although students felt that performance evaluations documented in their portfolio were fairly representative, they also perceived these to form a rather fragmented picture of their actual development. The portfolios provided snapshots rather than a complete picture of the student’s developmental trajectory. The portfolios mostly contained descriptions of single events concerning medical procedures and patient contacts that were observed because it was difficult for students to repeatedly collect performance evaluations of the same skills. Students only felt that the portfolio really reflected their competence development if they managed to collect feedback on the same task multiple times during their clerkship.

Students’ decisions about what evidence to upload to their portfolio were often determined by educational requirements concerning the WBAs content and frequency.

*Portfolio is of course, […] for me that is often just a lot of ticking off so it is very often a lot of things you have to ask […] So in my portfolio I think it is more meeting the requirements or the criteria, while here in the audio diary I just thought more like okay, what have I actually seen and done today and which experiences changed me or changed my way of thinking. (Student 18)*

Some experiences illustrative of their development were not part of the WBA requirements or simply not observed and therefore not documented in their portfolios. In their audio diaries students gave different examples of experiences that often were not documented: informal feedback, talks with peers, ethical dilemmas, mistakes, difficult situations, new experiences (e.g. first time taking a blood gas sample).

In addition, the content of the audio diaries and portfolios related mostly to the medical expert and communicator competency roles and, to a lesser degree, to the collaborator and professional roles. The remaining roles (i.e. health advocate, scholar) were hardly mentioned. Students commented that these underrepresented competency roles are often not explicitly addressed during the clerkships. They also mentioned not knowing what to include about these competencies in their portfolio because they did not have a clear idea of the content of the, in their opinion, less well-defined competency roles.

### Student beliefs about the purpose of a portfolio

Students had differing beliefs about the purpose of documenting information in their portfolio, which resulted in students including divergent experiences.

Some students predominantly considered a portfolio to be a tool to demonstrate progress and competence development. Therefore, these students were less inclined to document aspects in their portfolio that were, in their opinion, difficult to measure (e.g. self-confidence or assertiveness) or hard to show improvement in.

*It is something that is much less measurable and it is something that much less, well, that you can also concretely do much less about. And where you can show far less concrete improvements, because it is something that is in your head that you have to improve yourself […] But, well, how are you going to show a rising learning curve in asking for feedback? And how are you going to show a stronger learning curve for being confident. These are things you cannot assess. (Student 3)*

Other students perceived a portfolio to be more an instrument to document and demonstrate performance. Therefore, they were less inclined to document situations in which they had made a mistake, or moments when they had received critical feedback. Students feared that documenting these experiences in their portfolio would cause assessors to judge their performance as unsatisfactory. Also, documenting these perceived weaknesses would result in a lot of work because students have to follow-up on feedback, and provide evidence of improvement. Moreover, these students experienced their learning environment as competitive and were reluctant to ask for a WBA when they thought others had performed better. Students regretted these consequences of their mutual competition, though.

*In this clerkship, what I have noticed is that there is a lot pressure to get good assessments. And because other students have for instance received a very good assessment for something, you are going to think about it tactically, should I ask for something here, or should I not ask for something. And that’s a pity. (Student 11)*

### Student perceptions of the relevance of portfolio content

Students had various ideas about what information was relevant for their mentors and for portfolio assessors. Students were less inclined to share experiences that, in their opinion, concerned something that was predominantly relevant to them personally or part of their personality, but less relevant to their role as a physician. For example, when a student received feedback on her posture and non-verbal communication, she considered this to be something that was part of her personality and she had to work on this privately, not something to be shared with her mentor.

*Yes they did tell me to sit up straight […] that is something I often hear, also in other contexts so […] that’s something I have to work on on my own. (Student 4)*

Students preferred to document experiences in their portfolio that they thought would illustrate their unique, personal learning process. They did not document experiences when it concerned something that, in their opinion, all students had to go through, such as learning to combine work and private life. Although in their audio diaries students recognized that these experiences had influenced their competence development, they regarded these aspects as obvious and not worth mentioning in their portfolio.

### Student performance evaluation strategies

Students’ documentation of WBA feedback in their portfolio was influenced by their perceptions of feedback credibility. For example, students felt that they could only ask for a WBA when they had sufficiently contributed to the care of a patient and the supervisor had had ample opportunity to observe them through multiple direct encounters, because only then could the supervisor develop an accurate idea of their competence.

Also, students valued feedback on important steps in their development as explained by student 19:

*But I also ask for feedback especially when I have done something independently or I have done something new or I have done something differently or that I have been given feedback that I could not have thought of myself, that sort of thing. (Student 19)*

Moreover, students preferred to ask for a WBA from someone who they knew would provide detailed and useful feedback and if such a person was not present during an important learning experience, this experience was not documented.

### Portfolio structure

Students also described how the portfolio structure influenced how they documented their learning experiences.

Some experiences described in the audio diaries were difficult to capture in the portfolio. For example, conversations with faculty and peers had considerable impact on the student’s development. However, their portfolio did not include pre-structured forms which provide the opportunity to document these informal conversations, making it hard to document this information.

Furthermore, students would like to have more possibilities to provide their own perspective or reflection on WBAs captured in their portfolio. WBA forms did not contain textboxes for students to provide more details about the context in which an event took place. In the opinion of the students, adding this possibility would help others to better interpret performance data in the portfolio.

*That as a student you then don’t have any space in the portfolio to give your own opinion and to um to write what you think about that point of feedback and whether you agree with it […] and if you are going to do anything about it and if so, what you are going to do about it and um. […] So that the reviewer gets a bit better picture of how you yourself look at it. (Student 3)*

## Discussion

This study explored two questions:

1. How well do students think their portfolio reflects their competence development? and
2. How do students select and document their performance in a portfolio?

Students’ beliefs, their perceptions of relevant portfolio content, performance strategies and the portfolio assessment system influenced how, why and when they upload evidence on performance and development to their portfolios. These aspects influenced the extent to which the portfolio information accurately represented student performance and competence development. Overall, our findings suggest that a competency-based portfolio provides a fairly accurate, but fragmented picture of student development in clinical settings.

Our findings seem to confirm previous research on tensions between assessment for and of learning, and integrating both assessment purposes in portfolio use [12]. The students in our study who believed the portfolio’s main goal was to demonstrate performance tended to avoid documentation of critical feedback that reflected weaknesses and specific learning needs, as they feared that this might impact decisions about progress and achievement. As a consequence, meaningful feedback for learning is likely to be missed when reviewing portfolio information in mentor meetings. Bok et al. [12] also found that recording assessments in a portfolio was one of the reasons for students to perceive individual formative assessments as summative. This tension between learning and decision-making is problematic, as current educational approaches (e.g. competency-based medical education and programmatic assessment) use portfolios or WBAs for dual purposes [13–15]. The central idea behind the dual purpose of assessment is that assessment can be used to drive learning [16]. However, findings from our study seem to confirm that assessment can only drive learning when students feel safe to be vulnerable and disclose weaknesses they have to work on. Participants in our study indicated that they felt safer to document critical feedback in their audio diary as these data were not shared with their mentors and decision makers. The students in our study and several other studies thus sketch a clear picture: we are still far away from such a safe environment [12, 17]. In their insightful synthesis of the assessment literature, Watling and Ginsburg [18] propose ways to bridge the gap between the current assessment culture and learning environments that truly focus on the formative to ensure that learners are committed to continuous improvement. As summarized in one of their main conclusions “*We must embrace and routinely reinforce an improvement model of learning and of working, so that performing confidently is replaced by striving for improvement as a guiding professional value.”[p. 83].*

Findings from our study show that the non-medical expert CanMEDS roles were underrepresented in student portfolios. Students predominantly focused on the medical expert and communicator role and were less inclined to document progress on the other roles, e.g. professional and health advocate. Students commented that these underrepresented competency roles are often not explicitly addressed during clerkships and that they did not know what to include about these competencies in their portfolios. Rietmeijer and Teunissen [19] coined these underrepresented competencies as orphaned competencies. This underrepresentation of the non-medical expert roles is problematic, because both mentors and assessors need a complete and representative picture of the student’s competence development.

Our findings show that portfolios, by their very nature, result in fragmented documentation of student learning and performance. It illustrates the difficulties students may experience when trying to collect feedback during clerkships. Faculty’s lack of time and students’ reluctance to ask for feedback leads to feedback on isolated events rather than follow-up on feedback through repeated observation of clinical tasks. Some adaptations in our current WBA practice could support providing valuable feedback. For example, incorporating dedicated time for observation and feedback into the daily clinical program seems essential for promoting the exchange and documentation of feedback [20]. Moreover, videotaping consultations might enable supervisors to provide feedback when it fits their schedule [21]. Also, it is critical to find the right amount of WBAs. Less mandatory WBAs could result in more meaningful and higher quality WBA content. Students indicated that the high number of required WBAs combined with the busy workplace caused them to ask for feedback when it was easy, instead of valuable for their development. Moonen-van Loon, et al. [22] demonstrated that combining different WBA tools in a portfolio can lead to a more feasible amount of required WBAs while still allowing for reliable decision-making about resident performance.

Our study underlines the importance of involving student perceptions when designing portfolios. The students in our study expressed the need to have more freedom in their portfolios to express their perspectives and add comments clarifying characteristics of the learning context and assessment setting. They felt that this additional information would help assessors to develop a better understanding of their competence development. Captions could be used for this purpose [23]. Captions are textboxes attached to each portfolio document describing what the document is, why this is valuable evidence, and for what development it provides evidence [24]. That students need a more flexible portfolio resonates with the work of Van Tartwijk and Driessen [2], who argue that students should be provided with clear guidance on how to develop their portfolios, but should also be given room for describing their unique experiences and composing an authentic product. Students value experiencing some freedom to adjust the content of their portfolios to their personal preferences. Some students indicated that it was easier to document learning experiences and reflections using the audio diary than having to write it down. Using audio may therefore be a good alternative to written text in a portfolio. Besides providing more flexibility in students’ documentation of competence development, diaries may furthermore enhance learning and competence development by encouraging more frequent and timely reflection on recent performance feedback.

### Limitations

Several limitations must be mentioned. The assignment of keeping an audio diary is different from proving one’s competence in a portfolio. The two assignments will generate different kinds of responses. We must therefore be cautious about judgments based on such a comparison. The interviews with the students were important to help clarify the comparison.

We conducted this research at Maastricht University where a specific competency-based portfolio is used. Portfolios differ considerably in content and design. We advise replication of this study in other settings where different types of portfolios are used.

Moreover, it is possible that our participant sample consisted of mainly very motivated and high achieving students. Nine students decided to withdraw their participation because of the demanding clerkships. However, the portfolio and audio diary of the participating students showed that our sample did include students who struggled with their competence development during their clerkship.

## Conclusion

In clinical settings, a competency-based portfolio may provide a fairly accurate yet fragmented picture of student development. Non-medical expert roles tend to be underrepresented. This study confirms the importance of taking student perceptions into account when implementing a competency-based portfolio. Students would benefit from guidance on how to combine assessment and learning, and coaching on how to select and document their development in their portfolios. Flexibility in portfolio structure and requirements is essential to ensure optimal fit between students’ experienced competence development and portfolio documentation.

## Caption Electronic Supplementary Material

Appendix 1: Audio diary questions

Appendix 2: Interview guide
