# Supplementary material for: Student perspectives on competency-based portfolios: Does a portfolio reflect their competence development?
Source: Perspect Med Educ. 2020 Apr 9;9(3):166–72. doi: 10.1007/s40037-020-00571-7 (PMC7283408; doi:10.1007/s40037-020-00571-7)
Supplement: Supplementary file 1 — Appendix 1: Audio diary questions [file 40037_2020_571_MOESM1_ESM.docx]

# **Appendix 1: Audio diary questions**

1. What did you learn during the past days? Do you have examples?
2. Which experience made you discover how far along you are in your development? What did you discover about what you can and cannot do? Do you have examples?
3. What feedback did you receive that was valuable? What feedback made you discover how far along you are in your development?
4. Did you experience success during the past days? If yes, can you describe this experience?
5. Did you have a difficult experience during the past days? If yes, can you describe this experience?
