# Supplementary material for: Student perspectives on competency-based portfolios: Does a portfolio reflect their competence development?
Source: Perspect Med Educ. 2020 Apr 9;9(3):166–72. doi: 10.1007/s40037-020-00571-7 (PMC7283408; doi:10.1007/s40037-020-00571-7)
Supplement: Supplementary file 2 — Appendix 2: Interview guide [file 40037_2020_571_MOESM2_ESM.docx]

# **Appendix 2: Interview guide**

**Audio diary**

**1A00** You selected two types of learning experiences from your audio diary that were the most valuable in your opinion. Why did you select these experiences? Could you explain the difference between these experiences?

**1B00** Which learning experience you described in your audio diary was the most important for your learning process? Why this experience?

**1B10** Did you document this experience in your portfolio? Was this a deliberate choice?

**1B11** Do you think that the way in which this experience was documented in your portfolio provides valuable information about your development? Why?

**1C00** Which feedback you have documented in your audio diary was the most valuable for your learning process? Why this feedback?

**1C10** Did you document this feedback in your portfolio? Was this a deliberate choice?

**1C11** Do you think that the way in which this feedback was documented in your portfolio provides valuable information about your development? Why?

**Portfolio**

**2A00** Could you select two types of learning experiences from your portfolio that were the most insightful in your opinion. Why did you select these experiences? Could you explain the difference between these experiences?

**2B00** Do you think that the way in which these experiences are documented in your portfolio provides valuable information about your development? Why?

**2B10** Did you mention these experiences in your audio diary? Why?

**2C00** You also mention feedback/situations/learning experiences in your portfolio which you did not include in your audio diary. Could you elaborate on your reasons?

**2C10** What is your opinion about this information?

**2C20** To what extent is this information important for your mentor and portfolio assessors when assessing your development based on the performance documentation included in your portfolio?

**2D00** Is there anything that you would not include in your portfolio? Why not?

**2E00** What kind of performance documentation in the portfolio is not informative in your opinion?

**Current level of competence**

**3A00** Could you describe your level of competence for all the different competency domains?

**3B00** Did you feel competent during this clerkship?

**Portfolio and audio diary experiences**

**4A00** What is your opinion about the audio diary? What are your experiences with recording the audio diary?

**4B00** What is your opinion about the portfolio? What are your experiences with working on your portfolio?

**4C00** Did you work differently during this clerkship compared to how you worked during other clerkships? What is the difference? If yes, what are possible reasons for this difference in your opinion?

**4D00** What was the biggest difference between keeping your audio diary and working on your portfolio?
